# Reorganization of the Brain Extracellular Matrix in Hippocampal Sclerosis

**DOI:** 10.3390/ijms23158197

**Published:** 2022-07-25

**Authors:** Barbara Sitaš, Mihaela Bobić-Rasonja, Goran Mrak, Sara Trnski, Magdalena Krbot Skorić, Darko Orešković, Vinka Knezović, Željka Petelin Gadže, Zdravko Petanjek, Goran Šimić, Danijela Kolenc, Nataša Jovanov Milošević

**Affiliations:** 1 Department of Neurology, University Hospital Centre Zagreb, 10000 Zagreb, Croatia; barbara.sitas@gmail.com (B.S.); mkrbot@gmail.com (M.K.S.); zeljka.petelin@mef.hr (Ž.P.G.); 2 Department of Biology, School of Medicine, University of Zagreb, 10000 Zagreb, Croatia; mihaela.bobic@mef.hr; 3 Croatian Institute for Brain Research, Scientific Centre of Excellence for Basic, Clinical and Translational Neuroscience, School of Medicine, University of Zagreb, 10000 Zagreb, Croatia; strnski@hiim.hr (S.T.); vinka.knezovic@mef.hr (V.K.); zdravko.petanjek@mef.hr (Z.P.); gsimic@hiim.hr (G.Š.); 4 Department of Neurosurgery, University Hospital Centre Zagreb, 10000 Zagreb, Croatia; goran.mrak@gmail.com; 5 Department of Neurosurgery, Clinical Hospital Dubrava, 10000 Zagreb, Croatia; darkoreskov@gmail.com; 6 Department of Anatomy and Clinical Anatomy, School of Medicine, University of Zagreb, 10000 Zagreb, Croatia; 7 Referral Centre of the Ministry of Health of the Republic of Croatia for Epilepsy, Affiliated Partner of the ERN EpiCARE, 10000 Zagreb, Croatia; 8 Department for Neuroscience, School of Medicine, University of Zagreb, 10000 Zagreb, Croatia; 9 Lucerne Cantonal Hospital, Institute of Pathology, 6006 Lucerne, Switzerland; danijela.kolenc@gmail.com

**Keywords:** human hippocampus, drug-resistant epilepsy, perineuronal nets, aggrecan

## Abstract

The extracellular matrix (ECM) is an important regulator of excitability and synaptic plasticity, especially in its highly condensed form, the perineuronal nets (PNN). In patients with drug-resistant mesial temporal lobe epilepsy (MTLE), hippocampal sclerosis type 1 (HS1) is the most common histopathological finding. This study aimed to evaluate the ECM profile of HS1 in surgically treated drug-resistant patients with MTLE in correlation to clinical findings. Hippocampal sections were immunohistochemically stained for aggrecan, neurocan, versican, chondroitin-sulfate (CS56), fibronectin, Wisteria floribunda agglutinin (WFA), a nuclear neuronal marker (NeuN), parvalbumin (PV), and glial-fibrillary-acidic-protein (GFAP). In HS1, besides the reduced number of neurons and astrogliosis, we found a significantly changed expression pattern of versican, neurocan, aggrecan, WFA-specific glycosylation, and a reduced number of PNNs. Patients with a lower number of epileptic episodes had a less intense diffuse WFA staining in *Cornu Ammonis* (CA) fields. Our findings suggest that PNN reduction, changed ECM protein, and glycosylation expression pattern in HS1 might be involved in the pathogenesis and persistence of drug-resistant MTLE by contributing to the increase of CA pyramidal neurons’ excitability. This research corroborates the validity of ECM molecules and their modulators as a potential target for the development of new therapeutic approaches to drug-resistant epilepsy.

## 1. Introduction

Mesial temporal lobe epilepsy (MTLE) is the most common and best-defined form of symptomatic localization-related epilepsy, characterized by epileptogenic abnormalities in the limbic structures in the medial part of the temporal lobe [1]. The underlying pathological substrate is usually hippocampal sclerosis [2], and most patients show weak response or resistance to antiepileptic treatment. Due to its high incidence, MTLE is the most common form of drug-resistant epilepsy, and understanding its pathogenesis is crucial for developing new therapeutic strategies [3]. Numerous studies performed on surgically removed or autopsic human tissue (for review, see [4]) showed that dendritic sprouting, axon redistribution, and loss of parvalbumin neurons are the main findings in hippocampal sclerosis. Similar findings were reported in rodent models of MTLE [5,6,7], where significant attention was also dedicated to studying changes in the extracellular matrix [8].

Extracellular matrix (ECM) is an important regulator of excitability and synaptic plasticity, found to be altered in numerous psychiatric and neurological diseases [9,10]. ECM of the central nervous system (CNS) is heterogeneous and depends on the specific region, cell types, and subcellular domains that are incorporated into the ECM [11,12]. The main components of the neural ECM, synthesized and secreted by neurons and glial cells, are hyaluronic acid (HA, or hyaluronan) and proteoglycans of the lectican/hyalectian family, tenascins, and link proteins associated with them [13,14]. Perineuronal nets (PNN), abundant around parvalbumin expressing GABA-ergic interneurons, contain condensed, mesh-like, adult type of ECM that, during development, gradually replaces the juvenile, highly hydrated ECM. Once established, the composition of the mature ECM is mostly stable with little variation in their components, but this changes radically when lesions of adult CNS occur [13]. Previous studies showed how, following seizures in the mature CNS, the ECM composition is changed [8]. These changes include alterations in the expression of HA, neurocan, aggrecan, and other binding proteoglycans (PG) or glycoproteins, often degradation of all PNN structural elements with a concomitant increase of proteases activity [8]. Since seizures are a result of an imbalance between excitatory (primarily pyramidal neuron) and inhibitory (primarily GABA neuron) activity, changes in ECM composition around both excitatory and inhibitory neurons are thought to contribute to epileptogenesis [8]. For example, prolonged seizures cause synaptic rearrangements in the hippocampus, axonal sprouting, and increased dendritic spine length and number, processes that require permissive ECM [15]. Altered ECM around and in synapses might alter connectivity and functioning of inhibitory parvalbumin neurons activity, disturbing the balance between excitation and inhibition, and in the long run, can lead to pathological context. Elucidating the effects of seizures on the PNNs, ECM composition, its modulators, and vice versa will contribute to our understanding of how the extracellular environment contributes to epileptogenesis and may provide ground for research on future therapeutic targets.

## 2. Results

**Figure 1:**
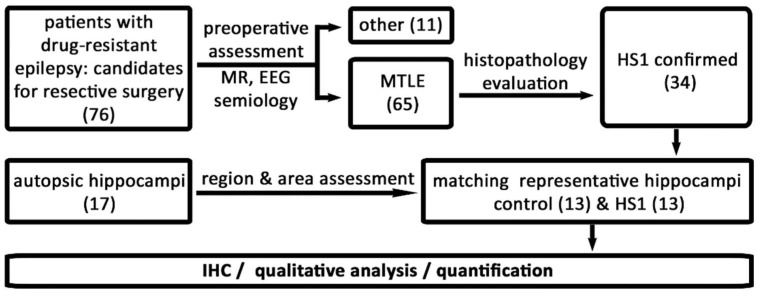
Study design. The study workflow presents the number (in brackets) of cases included throughout the study and further selected for neuropathological and immunohistochemical (IHC) analysis. Out of 34 selected blocks, 13 tissue blocks (38.2%) were both well preserved and had section cut orientation suitable for further analysis. For the IHC part of the study, these 13 HS1 and 13 autopsy tissue samples as control hippocampi were selected. The control group consisted of 13 samples of hippocampal tissue obtained during autopsy within 48 h post-mortem. A neurologist (BS), pathologist (DK), and neuroscientist with expertise in human brain histology (NJM) agreed on the selection of samples based on the suitability of the histological sections for all qualitative and semi-quantitative analyses. MTLE—mesial temporal lobe epilepsy; HS1—hippocampal sclerosis ILAE type 1; IHC—immunohistochemistry.

**Figure 2:**
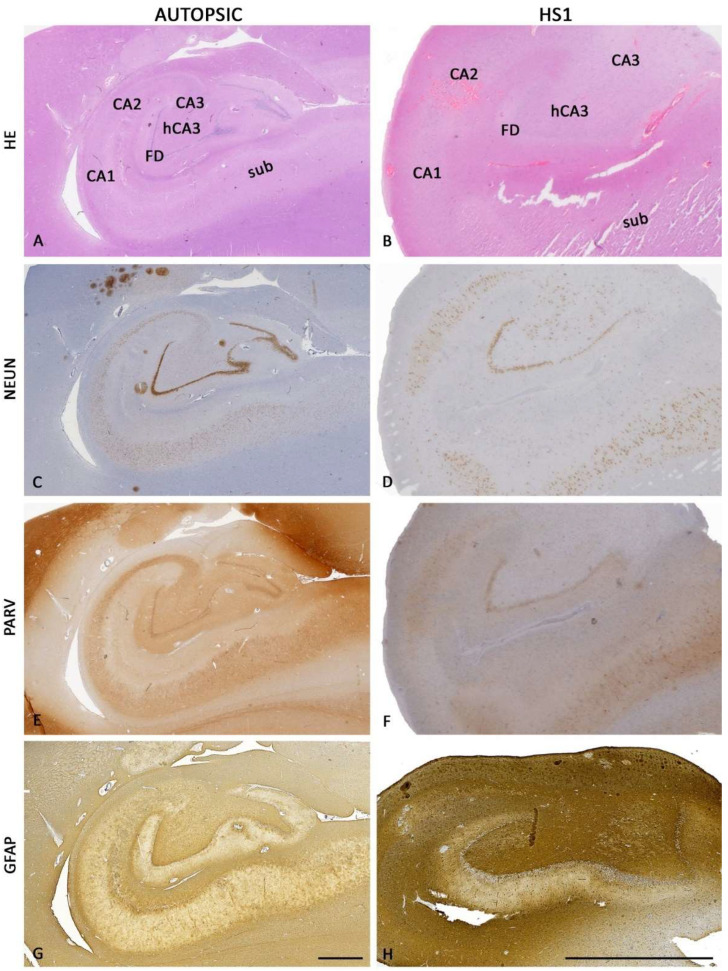
Coronal sections of human hippocampi: autopsic (left column of images (**A**,**C**,**E**,**G**)) and ILAE HS1 (right column of images, (**B**,**D**,**F**,**H**)) human hippocampi stained by hematoxylin-eosin (HE; (**A**,**B**)), for NeuN (**C**,**D**), parvalbumin (PARV; (**E**,**F**)) and glial fibrillary acidic protein (GFAP; (**G**,**H**)). HS1 is characterized by a generalized reduction of hippocampal size (in this case, closely to 3.5-fold). In addition, the loss of pyramidal neurons (**D**) and interneurons (**F**) is predominantly in the CA1 and hCA3 fields, and gliosis in all CA fields (**H**), which, together with the dispersion of the granular cell layer in the FD alters normal hippocampal histoarchitectonics in HS1. CA—Ammon’s horn fields; FD—fascia dentata; hCA3—hilar CA3 field; sub—subiculum. The magnification scale bar in (**G**) also refers to (**A**,**C**,**E**) and a length of 2 mm. H’s scale bar also refers to (**B**,**D**,**F**) and 2 mm length.

**Figure 3:**
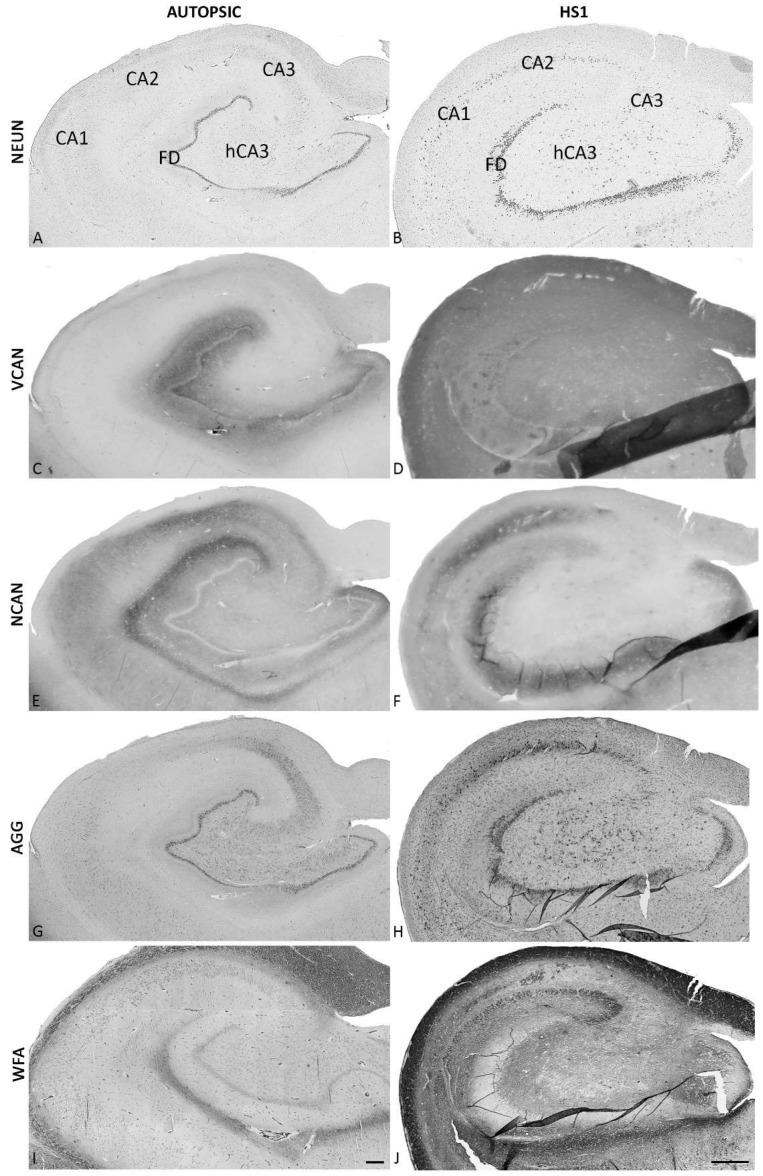
Coronal sections of human hippocampi: autopsic (left column of images, (**A**,**C**,**E**,**G**,**I**)) and sclerotic (HS1, right column of images, (**B**,**D**,**F**,**H**,**J**)) stained for NeuN (**A**,**B**), versican (VCAN; (**C**,**D**)), neurocan (NCAN; (**E**,**F**)), aggrecan (AGG; (**G**,**H**)) and with Wisteria Floribunda agglutinin (**I**,**J**). The VCAN exhibited the most prominent change of expression (**C**,**D**). While the diffuse VCAN expression was detected only in the FD of the autopsic hippocampi, a diffuse VCAN expression is prominent in all hippocampal fields of HS1 (**D**). On the contrary, the differential expression of NCAN among areas and layers of both autopsic hippocampi and HS1 (**E**,**F**) is slightly changed due to changed histoarchitectonics of the sclerotic hippocampi: a decreased NCAN expression is noticeable in CA3 areas while preserved in CA2 pyl, and robust in CA1′s mol (**F**). The condensed forms of the ECM are visualized by AGG and WFA (**G**,**J**) stainings of perineuronal nets (PNN) in CA’s pyl of the autopsic hippocampi as well as in the gcl and around solitary neurons in the polyml of the FD (**G**). In HS1, the AGG-ir is limited to subsets of neurons, mainly in gcl and pyl. Along with VCAN-ir, the most striking change in ECM organization is detected by WFA, indicating a change in the glycosylation pattern of proteoglycans and glycoproteins. The WFA staining is diffuse in HS1 (**J**) except CA1 mol, while in the FD and CA of autopsic hippocampi, it is reserved for perineuronal nets (**I**). For a detailed explanation of change in WFA expression, see text and Figure 6. Higher magnifications of the expression of diffuse ECM molecules and PNNs are presented in supplemental Figures S1–S3. CA—Ammon’s horn fields; FD—fascia dentata. The magnification scale bar in I indicates 500 μm and refers also to (**A**,**C**,**E**), and (**G**); the magnification scale bar in J indicates 500 μm and refers also to (**B**,**D**,**F**), and (**H**).

The pathohistological findings presented here corresponded well with the characteristic features of hippocampal sclerosis type 1 (HS1, according to the classification criteria of the International League Against Epilepsy (ILAE). The design of the study is briefly described in Figure 1. The pathohistological features include generalized atrophy of the hippocampus (Figure 2 and Figure 3), a lower density of pyramidal neurons predominantly in CA1 and hCA3 fields (Figure 2C,D and Figure 3A,B), accompanied by the dispersed (shorter in length but wider) *fascia dentata* (FD) granular cell layer (Figure 2 and Figure 3A,B,G,H), and consequent lower granule cell densities (Figure 2C,D). Except for the granular cell layer (gcl) and molecular layers (mol), delineating other layers in HS1 was challenging due to the neuronal loss (Figure 2C–F and Figure 3A,B), accompanied by severe astrogliosis (Figure 2G,H), which altogether distorted the normotypic hippocampal histoarchitecture.

### 2.1. Changed ECM Protein Constituents Expression Pattern in HS1

**Figure 4:**
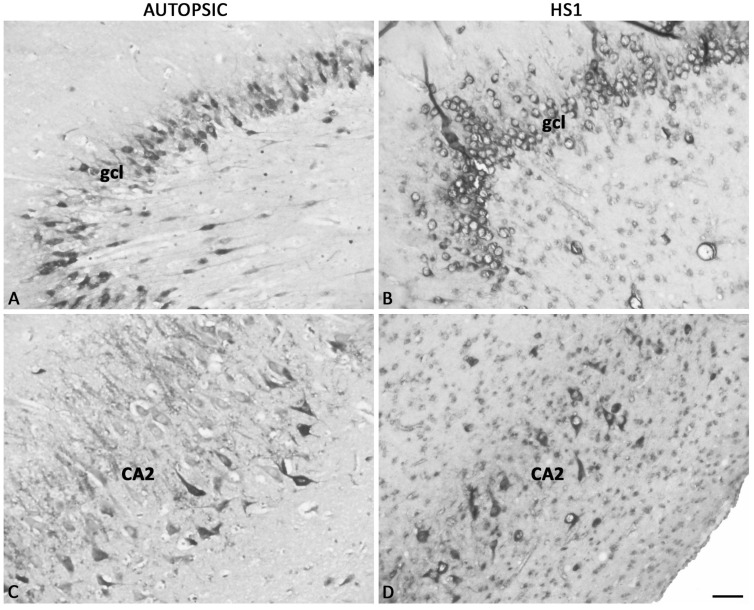
Immunohistochemically stained aggrecan (AGG) in sections of mature hippocampi autopsic (**A**,**C**) and HS1 (**B**,**D**). AGG-ir associated with condensed ECM is visible in the gcl and pyramidal layer of both autopsic hippocampi and HS1. AGG-ir in HS1 gcl surrounds round cells with less pronounced cell processes reflecting the changes in cell morphology in epilepsy. The same phenomenon is seen in the CA2 field, along with the loss of pale intracellular AGG-ir in sporadic cells of the pyramidal layer. CA—Ammon’s horn fields; gcl—granular cell layer. Higher magnification images of AGG expression in PNNs are presented in Supplemental Figure S2. The magnification scale bar represents 50 μm for (**A**–**D**).

**Figure 5:**
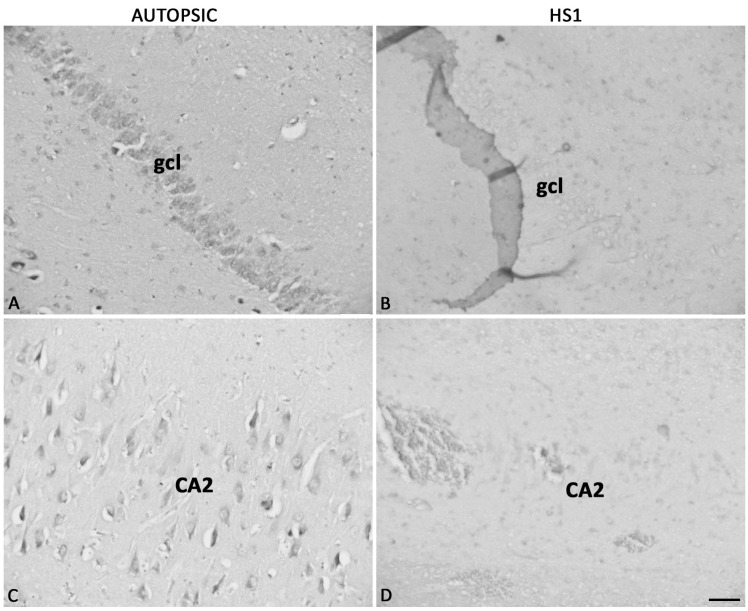
Pan-chondroitin-sulfate proteoglycan (CS-56) immunoreactivity in autopsic (**A**,**C**) and HS1 (**B**,**D**) hippocampi. The pan-chondroitin-sulfate proteoglycan CS-56 immunohistochemistry faintly marks perisomally expressed chondroitin-sulfate proteoglycans around the granular layer cells (gcl) of fascia dentata and the PNN in the CA of autopsic hippocampi that could not be distinguished in sections of HS1. CA—Ammon’s horn fields; gcl—granular cell layer. The magnification scale bar represents 50 μm and refers to (**A**–**D**).

Analysis of the sections stained with a panel of antibodies developed against extracellular molecular constituents shows a difference in expression patterns of all examined ECM-molecules between HS1 and autopsic hippocampi but to varying degrees (Figure 3C–J). The most striking change in the ECM molecules is the expression pattern of the proteoglycan versican (VCAN) (Figure 3C,D). The diffuse VCAN expression was detected only in the FD layers in the autopsic hippocampi. The highest expression was found in the polymorphic layer (polyml) with a decreasing gradient towards hCA3, somewhat lower expression in the FD-mol, and the weakest in the gcl (Figure 3C and Supplemental Figure S1). In HS1, a diffuse VCAN expression is prominent in all hippocampal fields without any gradual or differential expression related to a particular hippocampal area or layer (Figure 3D and Figure S1). Contrary to this uniform VCAN expression, the neurocan (NCAN) expression differs among areas and layers, and the area and layer differential expression pattern is present in the autopsic hippocampi and in HS1 (Figure 3E,F and Figure S1). A low expression level of NCAN is present in entire hippocampi sections, while some higher NCAN expression is evident in the mol and pyramidal layer (pyl) of CA, in particular in CA2 (Figure 3E and Figure S1). In HS1, the NCAN overall expression pattern is slightly changed due to changed histo-architecture of the sclerotic hippocampi: the expression of NCAN is decreased in the CA3 area while well preserved in CA2 pyl and increased in the mol of CA1 (Figure 3F and Figure S1). The aggrecan (AGG) expression in the mature hippocampus is related to the condensed form of ECM presented as perfectly formed PNNs in the pyl of the entire CA and sporadically around the solitary neurons in the polyml of FD (Figure 3G, Figure 4A,C and Figure S2). In HS1, the AGG expression loses its characteristic distribution to PNNs, and appearance is limited only to a subset of neurons. The AGG surrounds the majority of cells in gcl and pyl, delineating the change of their shape from triangular and fusiform to more round (Figure 4B,D and Figure S2). The pan-chondroitin-sulfate proteoglycan marker CS-56 shows discreet differences between autopsy and sclerotic hippocampi in its diffused and condensed expression pattern (Figure 5). In the autopsic hippocampi, CS-56 immunoreactivity (ir) is present very discretely in the gcl and as the CA PNNs (Figure 5A,C), whereas in HS1, no CS-56-ir was found (Figure 5B,D).

### 2.2. Changed Glycosylation Pattern of the Diffuse and Condensed ECM in HS1

**Figure 6:**
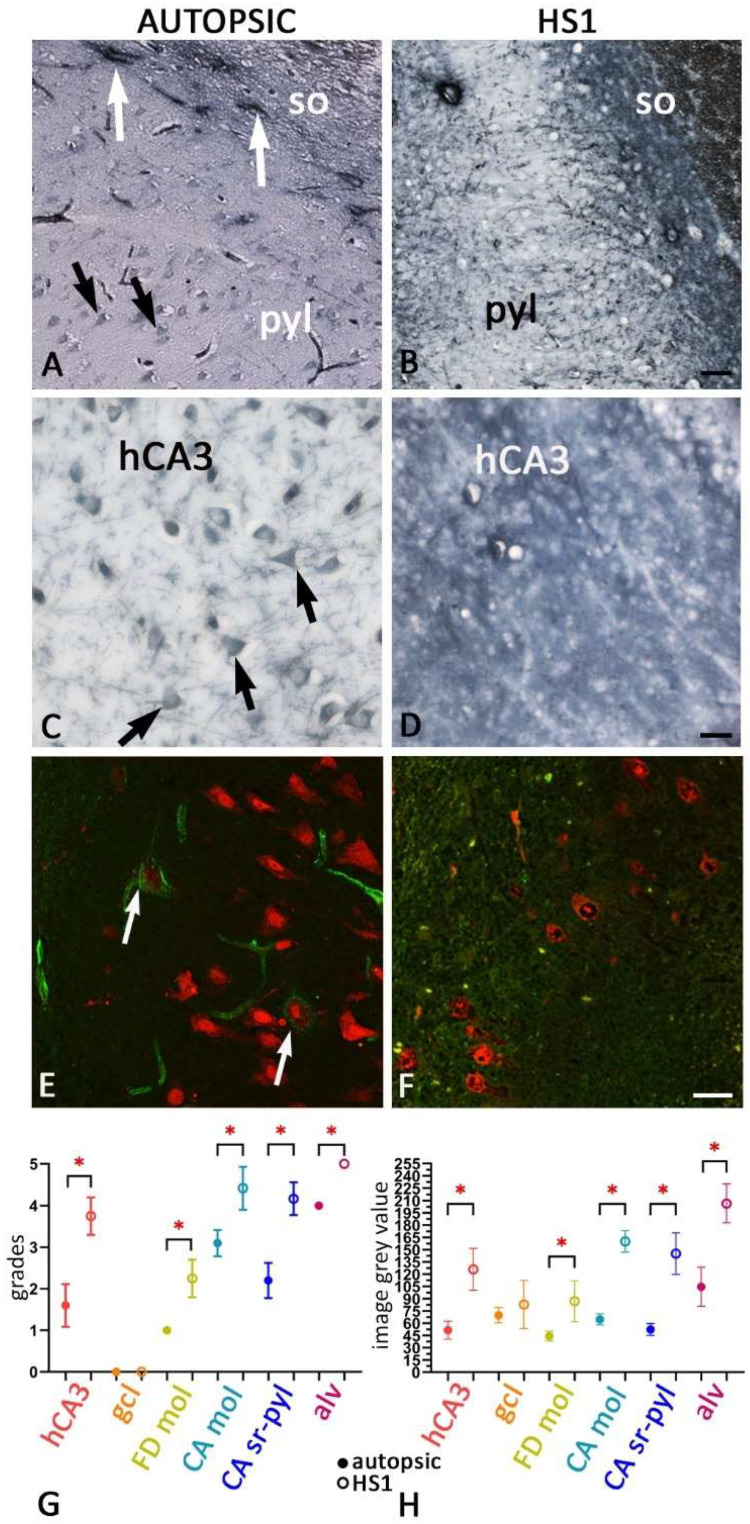
Changes in glycosylation patterns of ECM in HS1 shown by WFA staining (peroxidase and immunofluorescent labeling). The WFA staining of autopsic (**A**,**C**,**E**) and HS1 (**B**,**D**,**F**) hippocampal sections. In autopsic hippocampus (**A**,**C**,**E**), WFA positive PNN are seen around pyramidal neurons in the CA fields (arrows in (**C**,**E**)) and around non-pyramidal neurons, presumably PV interneurons in the CA1 stratum oriens (so, arrows in (**A**)). In HS1 subjects, there are only sporadically solitary PNN in the so, and a loss of pyramidal and non-pyramidal WFA-positive PNN (in other fields (**B**,**D**,**F**). An intense WFA staining of diffuse ECM is present in all hippocampal fields, most prominently in the alveus and the perforant pathway. A significant difference in WFA staining is seen between controls and HS1 across all hippocampal fields, with the positivity being more pronounced in HS1, inspected both by observers’ estimation (**G**) and using automated image analysis of microscopic slides (**H**). Hippocampal fields of the autopsic brains (dots represent medians, with range) showed an overall lower intensity of staining in comparison to HS1 hippocampal fields (empty dots represent medians, with range), with the difference being least prominent in the gcl. Higher magnification images of WFA-stained PNNs are presented in Supplemental Figure S3. CA—Ammon’s horn fields; so—stratum oriens; pyl—pyramidal layer; hCA3—hilar CA3 field; gcl—granular cell layer; FD—fascia dentata; mol—molecular layer; sr—stratum radiatum; alv—alveus. The magnification scale bars represent: 50 μm in (**B**) and also refers to (**A**); 20 μm in (**D**) and also refers to (**C**); 10 μm in (**F**) and also refers to (**E**). *—indicates a significant difference.

The second most striking change in the ECM in MTLE HS1 is the changed glycosylation pattern of the ECM molecules, as shown with WFA-specific staining (Figure 3I,J, Figure 6B–F and Supplemental Figure S3). The main finding in HS1 compared to autopsic hippocampi is an increased WFA staining of the diffuse ECM (Figure 3I,J, Figure 6B,D and Figure S3). In contrast, the specific WFA staining in the autopsic hippocampi is reserved for PNNs (Figure 3I and Figure 6A,C,E). The WFA primarily demarks PNNs around interneurons, pyramidal neurons, and a thin perisomatic area around granular cells in the autopsic hippocampi, while diffuse ECM lacks WFA staining (Figure 3I, Figure 6A,C,E and Figure S3). We found a significant increase of WFA staining (*p* < 0.01) of the diffuse ECM in HS1 across all fields when compared to autopsic hippocampal sections, as well as a more intensely stained alveus and perforant pathway (Figure 3J and Figure 6G,H). In some sections of HS1, solitary PNN can be seen situated in stratum oriens (so) of CA1-CA3, but not in pyl (Figure 6B,F) nor hCA3 (Figure 6D). Supplemental Table S1 shows data regards scoring the WFA staining used for semi-quantitative analysis. The polyml and mol of the FD in autopsic hippocampi are devoid of diffuse WFA-staining (Figure 3I). In some autopsic hippocampal slices, faintly WFA-stained fibers were seen within the superficial portion of CA mol, below the hippocampal sulcus (Figure 3I).

### 2.3. Correlation of Diagnosed Drug-Resistant MTLE-Related Clinical Data with the ECM Profile of HS1

In the next step, we compared the clinical data (age at onset, duration of epilepsy, number of antiepileptic drugs used one year before surgery, frequency of seizures one year before surgery, postoperative outcome) to the ECM proteins expression and glycosylation pattern. Although it was not possible to observe statistical significance for the correlations (due to a limited number of samples), we observed a trend that patients with a lower number of epileptic episodes had a lower intensity of diffuse WFA staining in stratum radiatum pyramidale in the CA fields. Since it is important to consider clinical data in the analysis of histological sections, in Supplemental Table S2, clinical data relevant to the excitation/inhibition imbalance are presented, with emphasis on the duration of disease, number of antiepileptic drugs, and comorbidities such as depression. The comorbidities often share similar underlying causes, such as neuroanatomical abnormalities and neurotransmitter imbalance.

### 2.4. Qualitative and Quantitative Changes in Drug-Resistant MTLE HS1 in Parvalbumin Interneurons and Astroglia

In our study sample, all autopsic hippocampal sections contained PARVir neurons, with an average of 5-fold more PARV-ir neurons per section (mean 36.6 per section), than in HS1 sections (mean 7.1 per section, *t*-test, *p* = 1.33 × 10^−13^). The significance of the lower number of neurons in HS1 is partially due to the complete absence of PARV-ir neurons in 20% of the HS1 sections. The most striking difference was in the CA1 field, wherein 60% of HS1 sections PARV-ir neurons were not observed (mean 0.7 per CA1 section), whereas in the autopsic CA1 field, an average of 5 neurons per section was found. In the HS1, the distinction of the layers of CA was not possible due to sclerosis, and therefore challenging to detect layer-specific changes. In sections of HS1, a notably reduced number of PARV-ir cells is seen in the FD granular layer, which still contains single PARV-ir neurons. In contrast, other layers of FD are utterly devoid of PARV-ir neurons. Supplemental Table S3 includes the data on the number of PARV+ neurons per region/layer for each analyzed section from autopsic and resected HS1 specimens.

**Figure 7:**
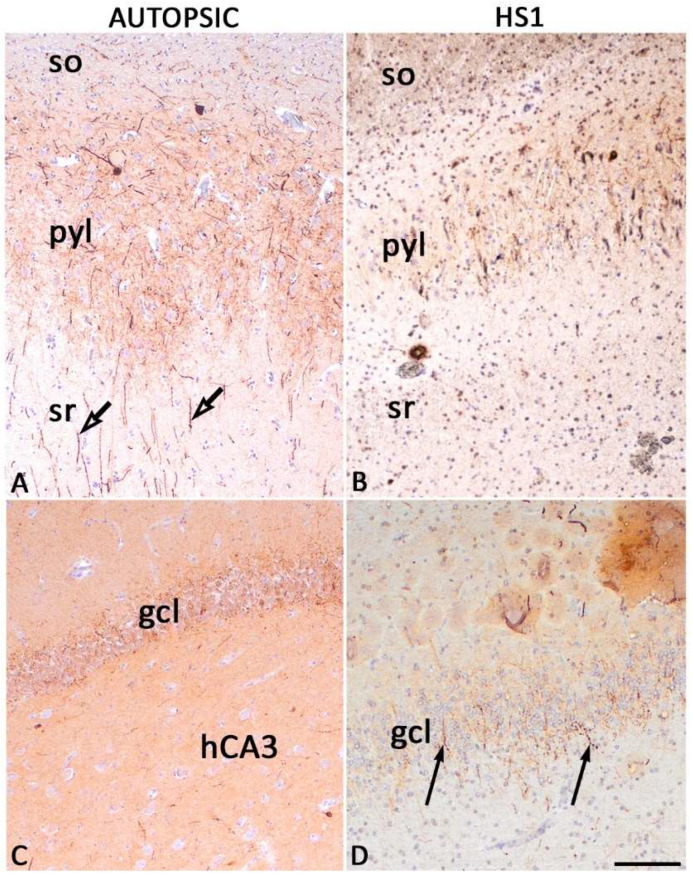
Comparison of parvalbumin expression in autopsic hippocampi (**A**,**C**) and HS1 (**B**,**D**). In comparison to autopsic hippocampi, parvalbumin-ir neurons in the pyramidal layer and stratum oriens in HS1 hippocampi are small, oval, and faintly stained (**A**,**B**). In autopsic hippocampi, the PARV-ir fibers are present around neurons in all CA fields, radially extending throughout the CA1 sr (arrows in (**A**)). The loss of parvalbumin-ir neuropil in the autopsic hippocampus (**B**) is seen throughout the hippocampus, with this difference being most prominent in the stratum radiatum (sr) and pyramidal layer (pyl) of the CA1 field. In the granule cell layer (gcl) of the fascia dentata, parvalbumin-ir neuropil is present mostly as punctiform staining of fibers or continuously stained fibers (**C**). In HS1 gcl (**D**), there were fewer punctiform stained fibers than in autopsic hippocampi, but those that remained stained through their long axis, with parallel orientation, are easily observable (arrows in (**D**)). so—stratum oriens; pyl—pyramidal layer; sr—stratum radiatum; hCA3—hilar CA3 field; gcl—granular cell layer. The magnification scale bar indicates 200 μm and also refers to (**A**–**C**).

The PARV-ir neurons in autopsic hippocampi were larger than in HS1 with sharply outlined triangular, fusiform, or multipolar shapes (Supplemental Figure S4A,C,E) in the pyl and few PARV-ir neurons per section in the CA so (Figure 7A,B). The PARV-ir fibers are present around neurons in all CA fields, radially extending throughout the CA1 sr (arrows in Figure 7A). Although the number of PARV-ir neurons in HS1 was spared to some extent, these neurons were small, oval, and faintly PARV-ir, with rarely visible dendrites (Figure 2, Figure 7 and Figure S4). While the overall loss of PARV-ir neuropil is evident throughout the entire hippocampus, the loss of PARV-ir neurites in the CA pyl was prominent (Figure 7B,D). In the FD gcl of HS1, the punctiform PARV-ir fibers were fewer than in autopsic FD, but the preserved fibers were clearly stained and easily observable (Figure 7C,D).

The severe gliosis in the HS1 samples was obvious in sections IHC stained against the glial-acidic-fibrillary-protein (GFAP) (Supplemental Figure S5). The radially oriented GFAP immunoreactive fibers, seen clearly in the FD’s ml in autopsic hippocampi (Figure S3A), are rarely visible in HS1 (Figure S5B). In HS1, the GFAP-ir cells and thin fibers are more pronounced in other layers and less in FD’s ml and gcl (empty blue arrows in Figure S5B). Therefore, the layer of astrocytes discernible between the gcl and polyml visible in autopsic hippocampi (black arrows in Figure S5A) is not observable in HS1.

**Figure 8:**
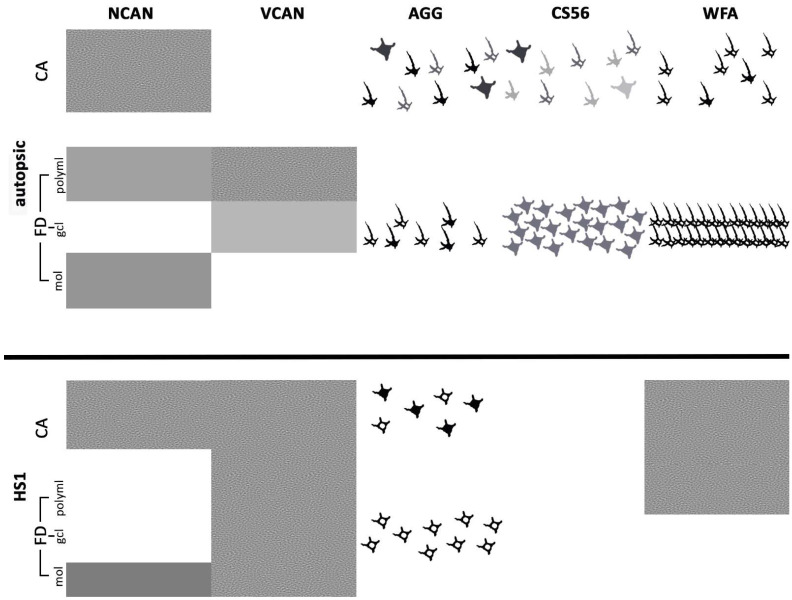
Schematic summary of the key differences in expression of ECM components between autopsic (**upper half**) and HS1 (**lower half**) hippocampi. The main change of neurocan expression is the diffuse-ir in the FD layers in HS1. Versican is expressed faintly diffusely in the gcl and polyml but firmly in all layers of the hippocampus. Note the reduction of aggrecan-ir cell density and the faint-ir of dendrites following HS1 compared to autopsic hippocampi. The CS56 staining is absent in HS1. The prominent change of glycosylation, shown by WFA staining, is the conversion of the extracellular staining associated with PNNs in autopsic specimens to the overall diffuse extracellular staining in HS1. Note the reduction in the size of HS1 hippocampal layers. Diffuse-ir of ECM components is noted with different shading of grey. Intracellular and extracellular expression of ECM components is presented by grey or black schematic drawings of cells and their processes. FD—fascia dentata; CA—Ammon’s horn field; mol—molecular layer; gcl—granular cell layer; polyml—polymorphic layer.

A summary of the found expression pattern of ECM molecules is presented with a schematic drawing in Figure 8.

## 3. Discussion

This work aimed to reveal the ECM profile of HS1 specimens that were surgically removed during the treatment of drug-resistant epilepsy. We evaluated the expression pattern of the molecules recognized as modulators of excitability and synaptic plasticity, some of which are constituents of the diffuse and condensed ECM. Results showed that in the sclerotic hippocampus, both forms of ECM, the condensed and the diffuse, are changed in a manner unfavorable for stabilization and maintenance of its functional connectivity, which is a prerequisite for healthy non-epileptogenic excitability of hippocampal circuits.

In brief, HS1 tissue displayed a decrease in condensed ECM organization, with changes in PNN’s composition and distribution. At the same time, an overall increase of diffusely expressed ECM constituents was present uniformly in all layers and areas of the HS1 hippocampi.

Different seizure presentations in experimental models and humans exhibit PNN disruption as a consequence of elevated protease activity. Recently it has been demonstrated that remodeling of PNNs is implicated in schizophrenia, traumatic brain injury, Alzheimer’s disease, and epilepsy [9,10,11,16,17,18,19]. Considering the ECM lecticans as the fourth component of the quadripartite synapse [20] and seizures as a result of an imbalance of synaptic activity of excitatory, primarily pyramidal principal neurons and inhibitory, primarily GABA interneurons, changes in expression of lecticans and altered binding to hyaluronan around these neurons could contribute to epileptogenesis [8]. Indeed, some previous studies in animal models showed how, following seizures in the mature CNS, the expression of neurocan, aggrecan, and hyaluronan is changed in such a way that a portion of PNN structures are degraded while protease activity is increased [8,21]. These alterations may contribute to the accumulation of free (unbound) hyaluronan, which favors neurite outgrowth and synaptic plasticity [8]. On the other end, the mature ECM, diffusible or condensed in PNNs, is primarily inhibitory to structural or activity-dependent plasticity. Knowing that, we questioned whether seizures coincide or provoke the expression of a different set of ECM molecules which leads the organization to a more immature type that supports reactivation of plasticity mechanisms following injury?

Therefore, we examined the juvenile V0 isoform of versican in the mature autopsic and HS1 samples and observed a changed expression pattern. In our study of the autopsic hippocampi, the V0 diffusible versican isoform expression is confined in FD, where the highest plasticity is present, which was expected, while other areas are free of this juvenile isoform. In HS1, contrary to this, V0 versican is upregulated in all layers and areas of the hippocampus. Thus, this observation confirms that in the HS1 in drug-resistant epilepsy, the juvenile (dV0) form of versican is broadly diffusely upregulated across all hippocampal fields. Similarly, neurocan, highly expressed in immature neural tissue, is expressed at a relatively lower level in CA1 and CA3 and to some higher degree in *stratum lacunosum* and CA2 pyramidal layer in autopsic mature hippocampi. This neurocan expression pattern is downregulated in all CA fields except the CA2 pyramidal layer and upregulated in the molecular layer in HS1.

Alterations in glycosylation patterns in proteoglycans and glycoproteins have been previously described as consequences of epileptic activity and etiological/risk factors [22,23,24]. In our study, the changed WFA staining pattern from that confined to PNNs into diffuse staining almost in all layers speaks in favor of global upregulation of HA and GalNAc glycosylation of proteoglycans and glycoproteins which is not surprising considering that, under normal circumstances, 98% of the total CSPG are present in the diffuse matrix, while only 2% within PNN(s) [16]. Altogether, this leads to assumptions that this pattern with a lack of PNN allows enhanced excitability and plasticity, similarly to that found in experimental models [8,16,23]. In fact, in our study, this difference in GalNac-specific glycosylation found in HS1 is statistically significant in the entire CA, and we observed a tendency for the lower intensity of diffuse WFA staining in patients with fewer seizure frequencies. This implies a more pronounced role of the glycosylation of diffused ECM in epileptogenesis. Increased GFAP reactive astrocytes are a typical finding after CNS injuries [24,25,26]. Strong astrogliosis in all fields in HS1 (except the FD molecular layer), which overlaps the expression pattern of WFA-specific glycosylation of the diffuse ECM, points to astrocytes as sources of ECM reorganization in epilepsy. Although ECM of both neuronal and glial origin is required for synapse formation and stabilization [27], glia alone is thought to be pro-excitatory [26].

Previous studies report that PNNs are formed predominantly around PARV interneurons and, to a lesser extent, pyramidal neurons (PyN) [16,19], but our results show that most PyN of autopsic CA have pronounced PNNs around them [28]. Our results also show a significant reduction of PyN/PNNs in CA1 in HS1. In addition, a significant reduction of PARV/PNN interneurons is evident in the HS1 CA1 field compared to autopsic tissue, while hCA3 PARV-ir neurons were, compared to other areas, preserved. According to some recent studies, PNNs are dynamic structures that may assemble and degrade in adult brains, too [23]. This gives us the possibility for speculations that in epilepsy, after degradation of PNNs, they should again develop and support new neuronal circuits. According to our results, in epilepsy, that is not the case since a smaller number of PNNs are found in cases of advanced MTLE.

Nevertheless, the PARV interneurons depletion is significant in our cohort. The loss of GABAergic neurons and reduced inhibitory activity was known to correlate with epileptogenic activity, although the direct causal link is still not fully understood [29]. The loss of the typical morphology and number of PARV neurons in the FD in MTLE has already been recognized [30,31,32,33], but there has been scarce evidence of other hippocampal fields. On the other side, as PNNs compartmentalize certain membrane proteins and ion channels directly affecting neurons’ activity [34], concomitant loss of PNNs modulators of synaptic activity in both pyramidal and PARV-ir interneurons may give a cumulative contribution to epileptogenesis.

The number of subjects analyzed quantitatively was defined by rigorous sample selection and study design (Figure 1). The sample selection aims to anticipate and neutralize cofounding factors, among other delays from tissue isolation to fixation that could decrease the accuracy of ECM molecules detection and consequently compromise the comparison of the autopsy vs. surgically obtained hippocampi. In addition, markers used to screen ECM changes were directed towards proteins’ epitopes that are not particularly sensitive to ex vivo time duration prior to fixation. Including cases with a longer delay to fixation to increase the total number of subjects would be counterproductive, in our opinion.

It would be beneficial to compare the ECM profile in subjects with drug-resistant epilepsy other than MTLE to elucidate the sequence of molecular and tissue changes in HS1. In particular, such analysis could help to understand whether this ECM reorganization is a consequence or part of the epileptogenic tissue changes. Unfortunately, our non-MTLE sample (11 subjects) was heterogeneous regarding pathology (3 temporal gangliogliomas, 2 dysembryoplastic neuroepithelial tumor—DNET, 3 malformations of cortical development, and 3 frontal focal cortical dysplasia). The second, even more limiting, factor with respect to comparability is the fact that isolated brain regions have different cytoarchitecture, quite distinct to hippocampal slices. In future studies, these pathological entities, in case of sufficient sample size, could be of crucial value in the research of epileptogenesis. Patients with brain tumors commonly have epileptic episodes. An experimental glioma (animal model with glioma grafts from patients) showed that tumor-released proteolytic enzymes degrade PNNs and their electrostatic insulation role, reducing specific membrane capacitance. This is proposed to allow fast-spiking neurons to exceed physiological firing rates [35].

Research on PNNs in neurodevelopmental disorders (among others, mainly schizophrenia) supports the hypothesis that abnormalities in constituents of PNN and ECM organization contribute to synaptic inhibitory insufficiency and synaptic instability, most likely in a region-specific manner, knowing the specific distribution of PNN and ECM molecules in cortical layers, regions or subcortically [18].

Compared to previous studies, our study has several advantages. It examines a higher number of MTLE patients, a more homogeneous group of subjects with diagnosed drug-resistant epilepsy, tissue intraoperatively isolated and immediately fixed, and finally, the ECM and cells were analyzed in all hippocampal fields. In addition, the knowledge of differences in the regional expression of distinct ECM components and distribution of PARV neurons potentiates the importance as well as the advantage of spatial-temporal matching of the hippocampal slices. Altogether, our qualitative and quantitative results support the idea that both condensed and diffuse forms of ECM and its glycosylation pattern have their role in drug-resistant epileptogenesis.

With this study, findings from experimental models of epilepsy, where reorganization of mature ECM organization towards juvenile ECM type is observed, were reevaluated, confirmed, and complemented in the human model, confirming the necessity and the translational value of these models. Furthermore, we convincingly showed the involvement of remodeled ECM in HS1 in patients with drug-resistant epilepsy, which was suggested by experimental modeling.

## 4. Materials and Methods

The study encompassed 76 samples of hippocampal tissue obtained during surgical treatment from subjects with confirmed drug-resistant epilepsy (the study design is presented in Figure 1). The subjects were preoperatively evaluated according to the standardized protocol [36] at the Department of Neurology, the Referral Centre of the Ministry of Health of the Republic of Croatia for Epilepsy, at the University Hospital Centre Zagreb. All clinical data from these patients were revised retrospectively and anonymized. Based on the clinical (semiology, EEG, MR) and histopathological assessment of hematoxylin-eosin (HE) sections by the pathologist (DK), 65 subjects had confirmed MTLE, while 11 samples had other causes of epilepsy and therefore were excluded from the histological part of the study (Figure 1). The exclusion criteria were based on found focal cortical dysplasia or other malformations of cortical development, or brain tumor in the temporal region. From the remaining 65 cases, in 34 patients, hippocampi were classified as hippocampal sclerosis type 1 (HS1) by ILAE (4). The fascia dentata (FD) and Cornu Ammonis (CA) fields (the CA1 to hCA3 areas) or their remnants were present in all HS1 samples. Control subjects were ante-mortem without neurological diseases or other conditions that would cause any possible changes in the hippocampi. The final selection of 26 samples (13 HS1 and 13 autopsic) was based on rigorous histological inclusion criteria. These criteria were: a clear presentation of the FD and the CA1–hCA3 fields on the comparable anterior-posterior levels of coronal histological sections (within the autopsic or HS1 group and between the groups) and, in the end, sex and age matching. After autopsy/surgery, the resected hippocampal tissue samples were conserved by immersion in 10% buffered formaldehyde for at least 48 h. The tissue was gradually dehydrated by immersion in ethanol solutions (70%, 80%, 96%, and 100%), at least 24 h per concentration. After that, tissue clearing was achieved by toluene for 6 h; tissue blocks were then immersed in melted paraffin at 60 °C for 48 h. The tissue blocks were cooled at 4 °C and serially cut into 5 µm (for HE staining) or 10 µm thin sections (for IHC) on a sliding microtome. Finally, the HE and indirect mono and double indirect light and fluorescent IHC staining were performed according to protocols described previously [37,38,39]. Briefly, after dewaxing with xylol, sections were rehydrated in a graded series of ethanol solutions, washed 3 × 10 min with Phosphate Buffered Saline (PBS), immersed in citrate buffer (pH 6.0), and heated in a microwave for 15 min at 90 °C for antigen retrieval. After washing, the sections were immersed in 0.3% hydrogen peroxide (in a 3:1 mixture of methanol and re-distilled water) for 30 min, washed in PBS, then incubated for 2 h in the blocking solution (PBS containing 5% bovine serum albumin and 0.5% Triton X-100, all from Sigma, St. Louis, MO, USA) at room temperature (RT) to prevent non-specific background staining. The primary and secondary antibodies and their dilutions used in this study are described in Table 1. The incubation with primary atb. lasted for 48 h at 4 °C, sections were then rinsed in PBS and further incubated with secondary atb. for 1 h/RT (Vectastain ABC kit; Vector Laboratories, Burlingame, CA, USA). Vectastain ABC reagents (streptavidin–peroxidase complex) were used in the subsequent step for 1 h/RT, rinsed in PBS for 3 × 10 min, and finally, peroxidase activity was visualized with 3,3′-Diaminobenzidine Peroxidase Substrate (Sigma-Aldrich, St. Luis, MO, USA). The sections were then rinsed with PBS, dried, cleared in Polyclear, and cover-slipped with Polymount (Polysciences Inc., Warrington, PA, USA). For the visualization of PNN, *Wisteria floribunda* agglutinin (WFA) was used instead of primary antibodies. The negative controls were included in all IHC experiments by (i) replacing the primary antibody with a blocking solution or pre-immune goat or horse serum or by (ii) omitting the secondary antibody or replacing it with an inappropriate secondary antibody.

The histological sections and images were analyzed using Olympus BX53 light or Olympus-IX83 microscope and Olympus UC-90 digital camera (Olympus Corporation, Shinjuku, Tokyo, Japan).

To qualify the HS1 sample group concerning the variability of pathological changes within, the three observers (BS, DK, NJM) independently performed a qualitative or semi-quantitative analysis of the specimens by rating the loss of neurons in the CA region and the dispersion of granular cell layer (gcl) of FD. The portion of NeuN-ir neurons in the CA1–hCA3 fields of HS1 sections was estimated by comparing the same areas (CA1–hCA3) in the autopsic sections. Compared to the total NeuN-ir neurons determined in the same fields in autopsic sections, the preservation of NeuN-ir in HS1 was evaluated and presented as a percentage. The PARV-ir neurons were counted per field (CA1, CA2, CA, 3, hCA3, and FD) for each HS1 and control section. The grade of dispersion of gcl was determined semi-quantitatively. A dispersion of the gcl in up to 20% of its presentation in the histological section was graded with “+”; dispersion between 21–50% with “++”, and dispersion more than 50% of its presentation on the slide with “+++”. The semi-quantification of WFA staining in autopsic and HS1 hippocampi was performed. Firstly, on high-resolution images of WFA-stained microscopic slides, areas of the hippocampus, such as FD—*stratum moleculare* (mol), CA3-1—*stratum radiatum* (sr), CA3-1—*stratum pyramidale* or pyramidal layer (pyl), CA3-1—*stratum oriens* (so), and fCA3 were delineated as regions of interest (ROI) using FIJI (40, version Java 1.8.0_172 (64-bit)) [40]. The 2 observers (BS and DK) independently assigned scores for each region of interest as follows: ‘0’ for none, ‘1’ for vague, ‘2’ for mild, ‘3’ for moderate, ‘4’ for severe, or ‘5’ for highest intensity. In addition, using FIJI [40], the intensity of WFA staining was measured, with the possible pixel value being 0 (not immunoreactive) or 255 (maximum immunoreactive staining) for each ROI. The median value for each histological area was calculated. Statistical analysis of diffuse ECM WFA positivity in controls and HS was performed using the Mann-Whitney test. Graphs were created using GraphPad Prism version 9.0 (GraphPad Software, San Diego, CA, USA).

Statistical analysis was performed using the IBM SPSS for Windows, version 25.0 software (IBM Corp., Armonk, NY, USA). Differences between the quantitative variables were tested with the non-parametric Mann-Whitney test. Association between variables was tested with Spearman’s correlation coefficient. *p*-values less than 0.05 were considered significant.

## 5. Conclusions

In conclusion, we showed the following changes in HS1 of drug-resistant MTLE: a decrease in condensed ECM organization, a disrupted PNN’s composition and distribution with loss of PNN around both pyramidal neurons and parvalbumin-containing interneurons, and simultaneously increased expression of diffuse ECM constituents with changed glycosylation pattern in all layers and areas of the hippocampi. Therefore, for a better understanding of epileptogenesis, and translation of basic discoveries into clinical practice, in addition to in vitro (slices and organoids) and animal models, further research on human material is needed. The ECM modulators are currently being investigated as potential treatment options. So far, the main complaint is that they have been shown to be inadequately selective. To find more selective ECM modulators or other potential therapeutic targets, knowledge of the changes in ECM composition in human brain tissue is a prerequisite. Additionally, in the coming era of personalized medicine, one of the goals should be to prevent the development of chronic, untreatable drug-resistant epilepsy in the early phase of the disease, where the ECM molecules are the critical factors of excitability and stabilizer of functional circuitry along with their enzyme remodelers that likewise show significant potential to be valid treatment targets.

## Figures and Tables

**Table 1:** List of primary and secondary antibodies.

Primary Antibody	Cat No.	Host, Isotype	Dilution	Supplier	Secondary Antibody
Anti-Neuron specific Nuclear protein (NeuN) AB 10711153	ab104225	Rabbit polyclonal IgG	1:1000	Abcam, Cambridge, UK	IHC: Rabbit, Vectastain ABC kit, PK 4001, USA IF: Donkey Anti-Rabbit 546, A10040, Thermo Fisher Scientific, Waltham, MA, USA
Anti-Glial Fibrillary Acidic Protein (GFAP) AB 10013382	GFAP (Z0334)	Rabbit polyclonal, purified immunoglobulin	1:1000	Dako, Glostrup, Denmark	Rabbit, Vectastain ABC kit, PK 4001, USA
Anti-Parvalbumin (PARV) AB 298032	Ab11427	Polyclonal rabbit	1:3000	Abcam, Cambridge, UK	IHC: Rabbit, Vectastain ABC kit, PK 4001, USA IF: Donkey Anti-Rabbit 546, A10040, Thermo Fisher Scientific, Waltham, MA, USA
Biotinylated *Wisteria floribunda* agglutinin (WFA) AB 2620171	L1516	N/A	6 µg/mL	Sigma Aldrich, Missouri, USA	N/A
Anti-CS-56 Anti-chondroitin sulfate AB 476879	C8035	Monoclonal mouse IgM	1:1000	SIGMA, St. Louis, MO, USA	Mouse, Vectastain ABC kit, PK 4010, USA
Anti-Fibronectin AB 476976	FN (F3648)	Polyclonal rabbit	1:400	SIGMA, St. Louis, MO, USA	Rabbit, Vectastain ABC kit, PK 4001, USA
Anti-Neurocan (NCAN)	HPA036814	Polyclonal rabbit	1:1000	SIGMA, St. Louis, MO, USA	Rabbit, Vectastain ABC kit, PK 4001, USA
*Wisteria floribunda* Lectin (WFA, WFL), Fluorescein AB 2336875	FL-1351-2	N/A	10 µg/mL	Vector Laboratories, Burlingame, CA, USA	N/A
Versican AB 2214378	AF3054	Polyclonal goat	2.5 µg/1 mL	Biotechne R&D Systems, Minneapolis, SAD	Goat, Vectastain ABC kit, PK 4005, USA
Aggrecan AB 90460	SAB4500662	Polyclonal rabbit	1:200	Sigma-Aldrich, Saint Louis, SAD	IHC: Rabbit, Vectastain ABC kit, PK 4001, USA IF: Donkey Anti-Rabbit 546, A10040, Thermo Fisher Scientific, Waltham, MA, USA

## Data Availability

Not applicable.

## Supplementary Materials

The supporting information can be downloaded at: https://www.mdpi.com/article/10.3390/ijms23158197/s1.

## Abbreviations

AGG	aggrecan
CA	Ammon’s horn
ECM	extracellular matrix
FD	*fascia dentata*
gcl	granular cell layer
GFAP	Glial Fibrillary Acidic Protein
HE	hematoxylin-eosin
HS1	hippocampal sclerosis type 1
IHC	immunohistochemistry
ir	immunoreactivity
mol	molecular layer
MTLE	mesial temporal lobe epilepsy
NCAN	neurocan
PBS	Phosphate Buffered Saline
PNN	perineuronal net
polyml	polymorphic layer
pyl	pyramidal layer
ROI	region of interest
RT	room temperature
so	*stratum oriens*
sr	*stratum radiatum*
VCAN	versican
WFA	*Wisteria floribunda* agglutinin
