# Supplementary material for: Reorganization of the Brain Extracellular Matrix in Hippocampal Sclerosis
Source: Int J Mol Sci. 2022 Jul 25;23(15):8197. doi: 10.3390/ijms23158197 (PMC9332352; doi:10.3390/ijms23158197)
Supplement: Supplementary file 1 [file ijms-23-08197-s001.zip › ijms-1804310-supplementary.pdf]

Supplemental figures

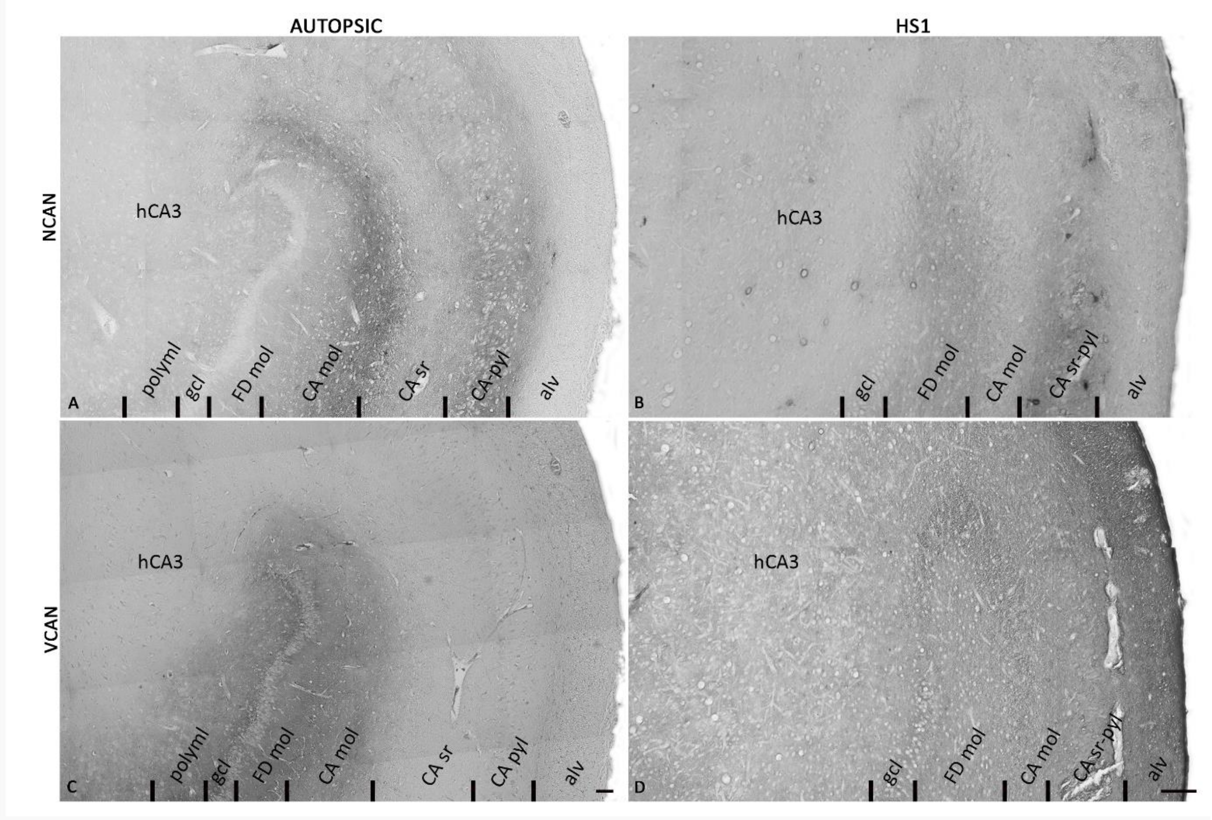

Supplemental Figure S1. High magnification images of NCAN and VCAN expression in FD and CA fields in autopsic hippocampus and HS1.

The NCAN and VCAN are expressed diffusely in all regions of hippocampi. NCAN expression in autopsic hippocampi is most prominent in the molecular and pyramidal layers of CA, and at a low level in all other layers except the granular cell layer (A). In HS1, due to gliosis and changes in overall histoarchitecture, layers are not easy to recognize and delineate. Still, the downregulation of the expression of NCAN in the molecular layer of CA and upregulation of expression in the molecular layer in FD is clear (B). VCAN is primarily expressed in FD in autopsic hippocampi, in molecular and in polyml layers, and at a very low level in the gcl (C). In HS1, VCAN is upregulated and expressed homogeneously through all layers and regions of hippocampi (D).

polyml- polymorphic layer, FD- fascia dentata, mol- molecular layer, gcl- granular cell layer, hCA3- hilar CA3 field, CA- *Cornu Ammonis,* alv-alveus, sr- stratum radiatum, pyl- pyramidal layer. The scale bars in C (refers also to A) and D (refers also to B) represent 100 μm.

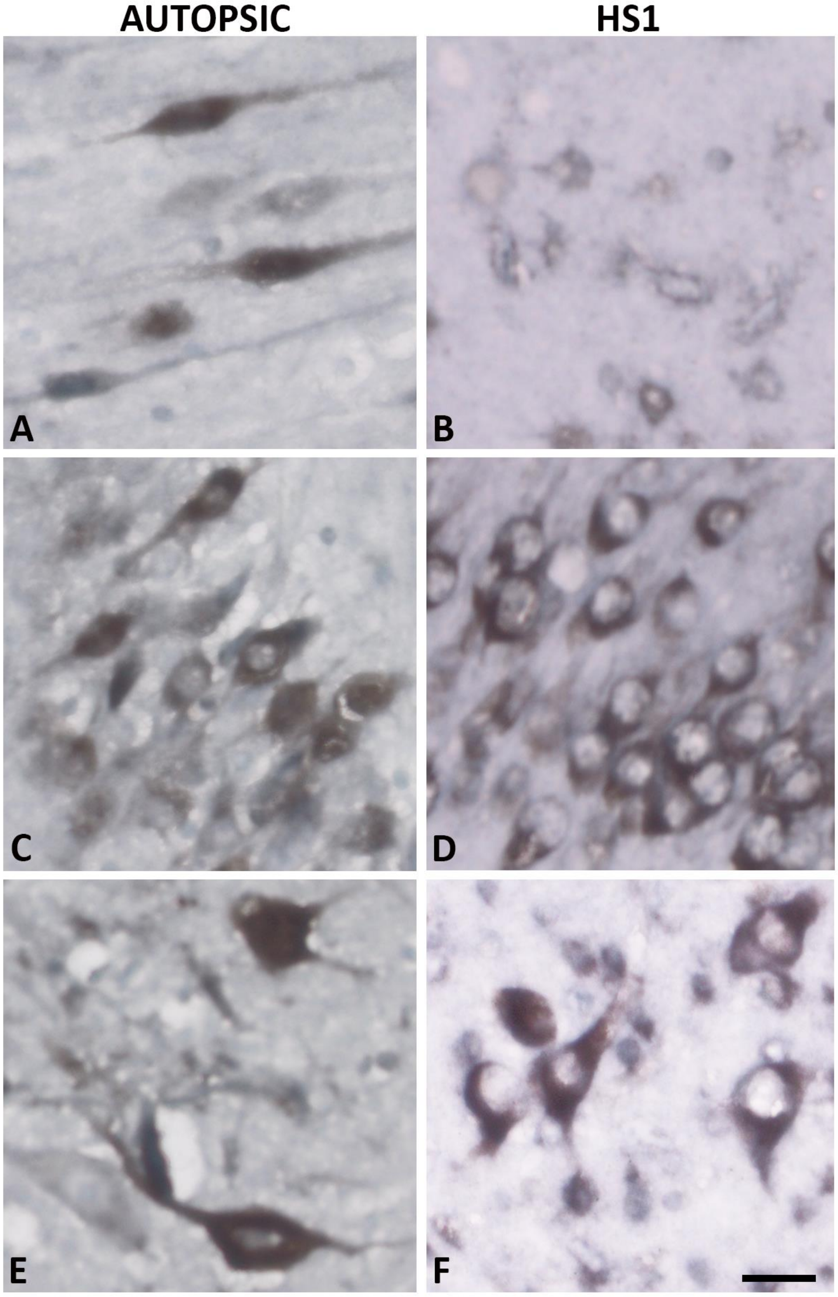

Supplemental Figure S2. High magnification images of AGG expression in the PNNs in the autopsic hippocampus and HS1.

The images of high magnification present AGG expression in PNNs of the autopsic hippocampus, in the polymorphic layer (A), around cell soma in granular cell layer (C), and the AGG expression in PNNs of the pyramidal layer of CA (E). However, the AGG expression in the HS1 is qualitatively different in these layers, we don’t find differentiated forms of PNNs, but predominantly an AGG expression around the cell soma in gcl and pyl, delineating the cell soma shape changes from triangular and fusiform to more round (B, D, and F). The magnification scale bar indicates 20 μm and refers to A-F.

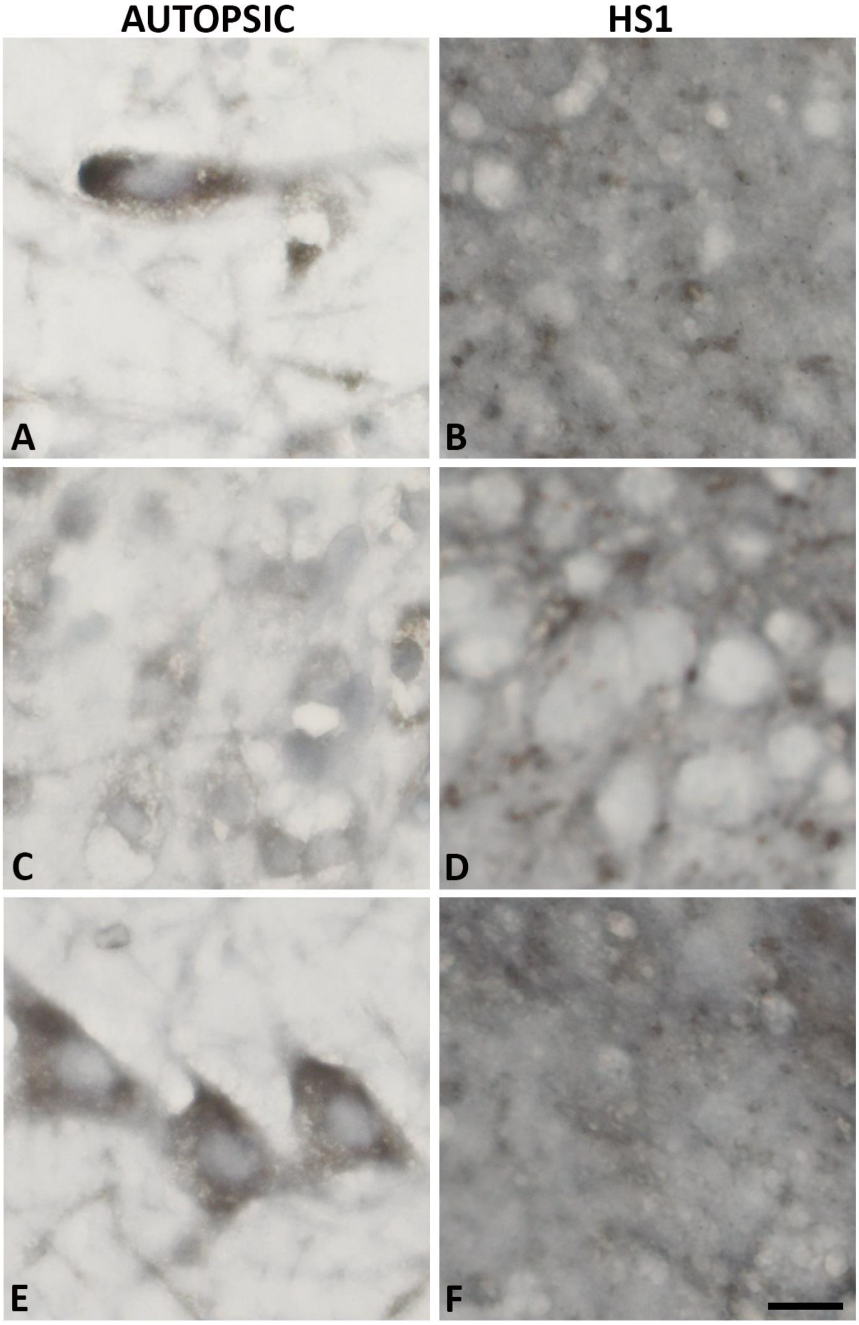

Supplemental Figure S3. High magnification images of WFA-specific staining of the PNNs in the autopsic hippocampus and WFA-specific staining of the diffusely expressed glycoproteins and proteoglycans in HS1.

The images of high magnification present WFA-specific glycosylation of glycoproteins and proteoglycans expressed in PNNs of the autopsic hippocampus, particularly in the polymorphic layer (A), and in the PNNs of the pyramidal layer of CA (E), while WFA staining is not observed in gcl (C). In the HS1, upregulation of WFA-specific glycosylation of diffusely expressed glycoproteins and proteoglycans is observed, and degradation or absence of PNNs (B, D, and F). The magnification scale bar indicates 20 μm and refers to A-F.

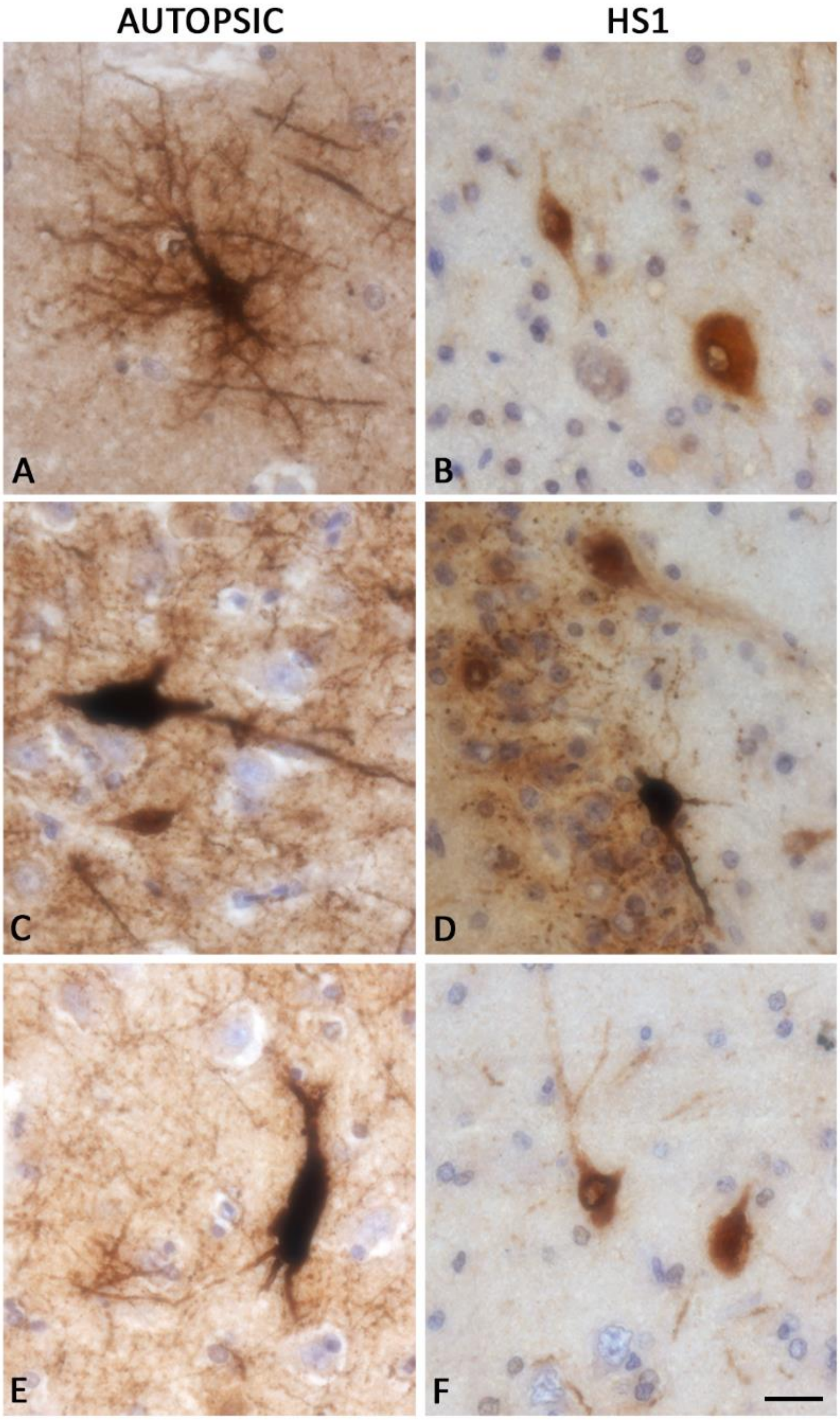

Supplemental Figure S4. Morphology of parvalbumin immunoreactive neurons in autopsic hippocampus and HS1.

Changes of neurons' morphology and PV-immunnoreactivity found in hippocampal sclerosis (right column of images) compared to controls (left column of images). The cell soma of PV neurons in pyramidal layer of autopsic hippocampi are sharply outlined and mostly pyramidal, but stellate, (A), bipolar (C), fusiform (E) are also present. In HS1, PARV-ir neurons are small, oval shaped, faintly ir, and their dendrites are rarely visible and regularly aspiny (B, D, F). The scale bar indicates 20 μm and refers to A-C.

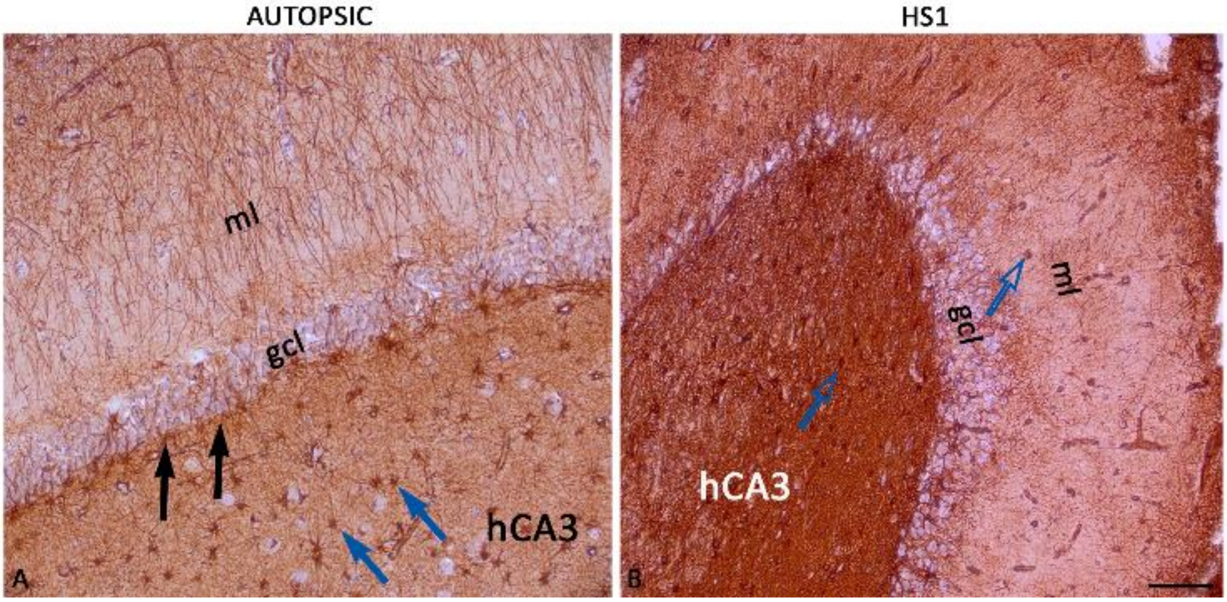

Supplemental Figure S5. Glial fibrillary acidic protein expression in autopsic hippocampus and gliosis in HS1.

GFAP expression in autopsic hippocampus (A) and HS1 (B). The severe diffuse reactive astrogliosis is best appreciated in the CA1 and hCA3 (B) fields of HS1 cases. The GFAP-immunoreactive fibers in this layer, seen in healthy hippocampi, are rarely visible in HS1 (A and B). The plexiform layer of FD consists of a single layer of astrocytes (black arrows in A), which is not discernible in HS1 (B). Reduced branching and size of astrocytes are seen in HS1 (empty blue arrows in B) compared to healthy controls (blue arrows in A).

GFAP- glial fibrillary acidic protein, mol- molecular layer, gcl- granular cell layer, hCA3- hilar CA3 field. The scale bar indicates 100 μm.

Supplemental Table S1. *Semiquantitative analysis of WFA staining intensity.*

Each row represents one microscopic slide of one specimen; numbers in brackets represent the scores for each region of interest: '0' for none, '1' for vague, '2' for mild, '3' for moderate, '4' for severe, or '5' for intense immunoreactivity.

	hCA3	FD mol	CA mol	CA sr-pyl	alv
**Autopsic**					
**1.**	2	1	3	2	4
**2.**	2	1	2	2	4
**3.**	2	1	3	2	4
**4.**	2	1	3	2	4
**5.**	1	1	3	1	4
**6.**	2	1	3	2	4
**7.**	1	1	3	2	4
**8.**	1	1	2	3	4
**9.**	1	1	3	2	4
**10.**	2	1	3	2	4
**HS 1**					
**1.**	3	2	4	3	5
**2.**	4	2	4	4	5
**3.**	4	3	4	3	5
**4.**	4	2	5	4	5
**5.**	3	2	4	3	5
**6.**	4	2	5	4	5
**7.**	3	2	5	4	5
**8.**	4	2	5	4	5
**9.**	4	3	5	4	5
**10.**	4	3	5	4	5
**11.**	4	2	4	2	5
**12.**	4	2	4	4	5

Supplemental Table S2. *Clinical details of HS1 patients.*

Abbreviations: *CBZ-CR-carbamazepine controlled released, LEV-levetiracetam, LTG-lamotrigine, CBZ-carbamazepine, OXC-oxcarbazepine, VPA-valproate, MPB-methyphenobarbital, CLB-clobazam, PRM-primidone, SUL-sultiame, TPM-topiramate, CNZ-clonazepam*

Clinical semiology: *all patients had typical clinical presentation of mesial temporal lobe epilepsy (MTLE) – focal (nonmotor seizures with impaired awareness) with evolution to bilateral tonic-clonic seizures. Considering detailed clinical semiology of focal seizures, 10 patients had focal autonomic impaired awareness seizures, 2 patients had focal cognitive impaired awareness seizures, and 1 patient had focal sensory impaired awareness seizure.*

SEX	Age at surgery (years)	Side	Epilepsy duration (years)	Febrile convulsion	Comorbidities	Clinical semiology	Number of antiepileptic drugs during disease course	Number of antiepileptic drugs 1 year before surgery	Old antiepileptic drugs 1 year before surgery	New antiepileptic drugs 1 year before surgery	Number of attacks before surgery	Post op. Outcome-Engel
Female	37	left	21	No	Depression	Epigastric aura followed by impaired awareness, speech arrest, postictal confusion	7	3	CBZ-CR	LEV, LTG	1 per month	1
Male	64	left	58	NA	Learning difficulties in school, Parkinson's disease	Epigastric aura, followed by impaired awareness and oroalimentary automatisms, speech arrest, postictal confusion	6	4	CBZ, VPA	OXC, LTG	Few every week	1
Male	42	left	37	NA	Depression	Epigastric aura, followed by impaired awareness and oroalimentary automatisms, speech arrest, postictal confusion	4	4	VPA, MPB	LTG, OXC	Few every/per week	2
Male	25	left	18	No	No	Epigastric aura, followed by impaired awareness and oroalimentary automatisms, speech arrest	NA	4	CLB, PRM, VPA	OXC	1 per month	1
Female	42	right	32	NA	No	Olfactory aura (hallucinations), followed by impaired awareness, and gestural automatisms with the upper limbs	NA	2	SUL	OXC	2-3 per month	1
Female	38	left	33	Yes	No	Epigastric aura followed by impaired	7	4	CBZ-CR, CLB	LTG, LEV	2-3 per month	1

						awareness and speech arrest						
Female	57	right	45	No	No	Focal cognitive (déjà-vu) impaired awareness seizure, gestural automatisms with the upper limbs	9	2	MPB	OXC	More than 1 per week	1
Female	32	right	22	No	Perinatal asphyxia, Speech difficulties in childhood, Depression	Epigastric aura, followed by impaired awareness and oroalimentary automatisms	6	4	MPB, CLB	LTG, OXC	daily	2
Male	42	left	10	Yes	Developmental psychomotor delay, Autism spectrum disorder	Epigastric aura, followed by impaired awareness and oroalimentary automatisms, speech arrest, postictal confusion	7	4	MPB, VPA	LEV, TPM	2-3 per month	1
Male	49	right	24	No	No	Epigastric aura, followed by impaired awareness and oroalimentary automatisms, postictal confusion	5	3	VPA	OXC, LTG	1 per week	1
Female	34	left	32	Yes	No	Epigastric aura, followed by impaired awareness and oroalimentary automatisms, speech arrest, postictal confusion	6	4	MPB, CNZ	OXC, LTG	1-2per week	1
Male	24	left	22	No	No	Epigastric aura, followed by impaired awareness and oral automatisms, postictal confusion	6	3		LEV, LTG, OXC	1 in a few months	1
Male	45	left	37	No	No	Focal cognitive (déjà-vu) impaired awareness seizure, with oral automatisms, speech arrest	6	4	MPB	LEV, OXC, LTG	2-3 per month	1

Supplemental Table S3a*. List of autopsic specimens used for quantification of PARV+ neurons and the number of PARV+ neurons across hippocampal areas.*

*Below, each microscopic slide is listed under the ordinal number. Numbers in cells represent the number of PARV+ neurons in different slides (rows) across hippocampal regions (columns). *N/A- not applicable*

**Number of PARV+ neurons**	hCA3	gcl	FD-mol	FD-polyml	CA3 - pyl	CA2-pyl	CA1 - pyl
**1.**							
A1-2	3	0	1	6	1	2	4
A1-6	3	3	0	16	0	1	2
A1-10	3	3	3	6	2	2	7
**2.**							
A7-3	1	0	1	4	0	2	3
A7-7	3	3	2	5	3	4	5
A7-11	3	1	5	7	4	2	3
**3.**							
A1-3	3	2	3	14	2	6	7
A1-7	3	5	0	13	2	2	6
A1-11	4	0	4	14	4	5	4
**4.**							
A2-2	7	2	5	20	2	2	16
A2-6	5	8	3	19	4	2	12
A2-10	9	10	6	20	4	4	9
**5.**							
A18-12	0	0	0	4	1	4	5
A18-11	0	1	0	2	1	3	4
**6.**							
B1-2	10	5	5	6	3	5	9
B1-6	5	4	1	6	3	1	12
B1-10	8	5	2	11	2	3	15
**7.**							
A15-2	6	2	5	28	6	1	17
A15-6	14	0	7	22	5	8	13
A15-10	10	0	5	40	6	3	13
**8.**							
A4-3	0	2	0	6	1	3	13
A4-7	1	1	0	11	5	6	12
A4-11	3	0	1	11	2	2	20
**9.**							
B1-2	0	1	0	14	N/A	N/A	2
B1-6	3	0	1	8	4	0	0
B1-10	5	0	1	11	2	2	2
**10.**							
B6-2	2	1	2	4	1	1	4

***Supplemental Table S3b**. List of HS1 specimens used for quantification of PARV+ neurons and the number of PARV+ neurons across hippocampal areas.*

*Below, each microscopic slide is listed under the ordinal number. Numbers in cells represent the number of PARV+ neurons in different slides (rows) across hippocampal regions (columns). *N/A- not applicable*

**Number of PARV+ neurons**	hCA3	gcl	FD-mol	FD-polyml	CA3 - pyl	CA 2-pyl	CA1 - pyl
**1.**							
7	5	0	0	0	3	0	0
11	5	3	0	0	1	0	0
15	3	0	0	0	0	0	0
**2.**							
1	0	0	0	0	0	0	0
5	0	0	0	0	1	0	0
9	0	0	0	0	0	0	0
13	0	0	0	0	0	0	0
17	0	0	0	0	0	0	0
**3.**							
13	7	10	8	0	3	1	3
17	15	5	6	0	3	5	7
7	7	0	6	0	0	0	3
11	8	2	3	0	N/A	N/A	4
15	7	2	4	1	3	3	5
19	8	1	6	0	0	0	0
**4.**							
7	5	1	0	0	0	0	0
8	2	1	0	0	0	0	1
20	1	1	0	0	0	0	1
**5.**							
1	1	2	0	0	0	0	1
9	0	4	0	0	0	6	0
8	0	1	0	0	3	N/A	0
19	0	3	0	0	0	0	0
**6.**							
1	5	0	0	0	0	2	0
5	10	0	0	0	0	0	0
9	6	1	0	0	0	2	1
13	4	0	0	0	0	0	0
17	4	2	0	0	0	0	0
**7.**							
1	3	0	0	0	N/A	N/A	N/A
9	1	0	0	0	2	N/A	N/A
17	0	0	0	0	N/A	N/A	N/A
8	N/A	N/A	N/A	N/A	N/A	N/A	N/A
**8.**							

9	0	0	1	0	3	0	1
7	4	1	0	0	0	0	1
15	5	2	0	0	1	0	0
**9.**							
1	4	3	2	2	1	1	1
5	3	1	0	0	N/A	N/A	N/A
13	5	9	0	0	N/A	N/A	5
17	1	4	0	0	N/A	N/A	2
11	N/A	N/A	N/A	N/A	N/A	N/A	N/A
**10.**							
1	0	0	0	0	0	0	0
17	0	0	0	0	0	0	0
16	0	1	0	0	0	0	0
**11.**							
1	4	3	1	0	0	0	0
5	3	0	0	0	1	0	1
13	0	1	0	0	0	0	0
17	2	0	0	0	1	N/A	N/A
**12.**							
7	2	0	0	0	8	10	0
